# Co-Production of a Flexibly Delivered Relapse Prevention Tool to Support the Self-Management of Long-Term Mental Health Conditions: Co-Design and User Testing Study

**DOI:** 10.2196/49110

**Published:** 2024-02-23

**Authors:** Alyssa Milton, Ingrid Ozols A M, Tayla Cassidy, Dana Jordan, Ellie Brown, Urska Arnautovska, Jim Cook, Darren Phung, Brynmor Lloyd-Evans, Sonia Johnson, Ian Hickie, Nick Glozier

**Affiliations:** 1 Faculty of Medicine and Health The University of Sydney Camperdown Australia; 2 Brain and Mind Centre University of Sydney Camperdown Australia; 3 ARC Centre of Excellence for Children and Families over the Life Course Sydney Australia; 4 mentalhealth@work (mh@work) Melbourne Australia; 5 Faculty of Medicine Nursing and Health Sciences Monash University Melbourne Australia; 6 One Door Mental Health Sydney Australia; 7 School of Social Work and Arts Charles Sturt University Canberra Australia; 8 Orygen Parkville Australia; 9 Centre for Youth Mental Health University of Melbourne Melbourne Australia; 10 Faculty of Medicine The University of Queensland Woolloongabba Australia; 11 TechLab ICT University of Sydney Sydney Australia; 12 Division of Psychiatry University College London London United Kingdom

**Keywords:** self-management, serious mental illness, self-care, digital health tools, blended interventions, peer support, mobile phone

## Abstract

**Background:**

Supported self-management interventions, which assist individuals in actively understanding and managing their own health conditions, have a robust evidence base for chronic physical illnesses, such as diabetes, but have been underused for long-term mental health conditions.

**Objective:**

This study aims to co-design and user test a mental health supported self-management intervention, My Personal Recovery Plan (MyPREP), that could be flexibly delivered via digital and traditional paper-based mediums.

**Methods:**

This study adopted a participatory design, user testing, and rapid prototyping methodologies, guided by 2 frameworks: the 2021 Medical Research Council framework for complex interventions and an Australian co-production framework. Participants were aged ≥18 years, self-identified as having a lived experience of using mental health services or working in a peer support role, and possessed English proficiency. The co-design and user testing processes involved a first round with 6 participants, focusing on adapting a self-management resource used in a large-scale randomized controlled trial in the United Kingdom, followed by a second round with 4 new participants for user testing the co-designed digital version. A final round for gathering qualitative feedback from 6 peer support workers was conducted. Data analysis involved transcription, coding, and thematic interpretation as well as the calculation of usability scores using the System Usability Scale.

**Results:**

The key themes identified during the co-design and user testing sessions were related to (1) the need for self-management tools to be flexible and well-integrated into mental health services, (2) the importance of language and how language preferences vary among individuals, (3) the need for self-management interventions to have the option of being supported when delivered in services, and (4) the potential of digitization to allow for a greater customization of self-management tools and the development of features based on individuals’ unique preferences and needs. The MyPREP paper version received a total usability score of 71, indicating C+ or *good* usability, whereas the digital version received a total usability score of 85.63, indicating A or *excellent* usability.

**Conclusions:**

There are international calls for mental health services to promote a culture of self-management, with supported self-management interventions being routinely offered. The resulting co-designed prototype of the Australian version of the self-management intervention MyPREP provides an avenue for supporting self-management in practice in a flexible manner. Involving end users, such as consumers and peer workers, from the beginning is vital to address their need for personalized and customized interventions and their choice in how interventions are delivered. Further implementation-effectiveness piloting of MyPREP in real-world mental health service settings is a critical next step.

## Introduction

### Background

Serious mental health conditions, including schizophrenia, bipolar disorder, and major unipolar depression, are associated with longer term use of mental health services [1]. Despite this substantial need, service provision is limited by funding and workforce constraints, and additional ways of supporting individuals with serious mental ill-health are required. Self-management programs have been developed to assist individuals with serious mental health conditions to actively understand and manage their own health [2]. Core components of self-management include psychoeducation, relapse prevention; the identification and avoidance of stressors; the development of effective coping strategies; and, often, a recovery element [3,4]. There is now substantial meta-analytic evidence that the provision of supported self-management programs (ie programs with guidance from a health professional or another helper) alongside standard care improves outcomes for people experiencing serious mental health conditions, including significant reductions in symptom severity, shorter length of admission, improved functioning, and better quality of life [4].

As a result of this robust evidence base [4], global health policies increasingly emphasize the importance of self-management interventions to support individuals with severe mental illness in managing their own health [5,6], and they are now included as a best practice recommendation in clinical guidelines (eg, the United Kingdom’s 2014 National Institute for Health and Care Excellence (NICE) Guidelines for Schizophrenia [7]). Despite this recommendation, self-management interventions are not always offered as a standard in services for people experiencing serious mental ill-health [4,8]. This is in contrast to services for people experiencing chronic physical illnesses, such as diabetes [9], where self-management programs are a core component of routine practice. Overall, self-management programs have the potential to benefit individuals experiencing serious mental ill-health and reduce strain on health services and have an economic rationale for reducing treatment costs [10], but implementation challenges remain.

Self-management programs typically involve the use of a collaborative learning process that encourages people experiencing serious mental ill-health to become experts in their own recovery [2,11]. Evidence suggests that supported self-management programs, with guidance from a health professional or another helper, are preferable to independent self-management for people with serious mental health conditions [2,12]. Self-management programs in which support is provided by a peer support worker (PSW) who has experienced a mental illness themselves have demonstrated promising evidence of effectiveness [12-15].

An example of a program that showed effectiveness was the Crisis Resolution Team Optimisation and Relapse Prevention (CORE) peer-supported self-management program [2,12,16], which was implemented in a large-scale (N=441), UK-based, randomized controlled superiority trial. This trial found that peer support using a structured workbook aimed at helping consumers develop self-management strategies to support their recovery beyond the immediate crisis led to a significantly lower rate of readmission to acute care within 1 year compared with self-management alone (29% for peer-facilitated self-management vs 38% for self-management control) [12]. Replication of the CORE study findings in routine settings across the UK National Health Service services and internationally has the potential to substantially reduce the burden on the acute care system. However, it is recognized that different countries may have specific nuances based on their cultures and systems [17,18]. As such, optimizing the delivery of the intervention in these different settings would benefit from consumer-driven consultation and co-production to translate the consumers’ needs into intervention components and refine the intervention accordingly before any research and implementation occur.

The peer-supported self-management intervention used in the CORE trial was systematically adapted in a stepwise co-production and piloting process in partnership with PSWs [2] from an existing recovery resource developed by Julie Repper, Miles Rinaldi, and their colleagues in South West London [19]. This existing paper-based resource “Taking Back Control” was itself co-produced with people with lived experience expertise; has a strong recovery focus; and incorporates self-management tools, including relapse prevention planning, goal setting, wellness planning, and a component for recovery from a mental health crisis [2].

Since the original resource was developed in 2007, the proliferation and advancement of technology, particularly smartphones, have provided an opportunity for digital interventions to become more accessible and acceptable to people experiencing serious mental ill-health [20]. People experiencing psychosis have adopted digital technology comparably to the general population [21], and mental health interventions delivered via smartphones are acceptable and feasible for people with psychosis and have the potential to support recovery [22-24]. However, access to technology has been found to vary among people experiencing schizophrenia by age, and a proportion do not use technology to manage their condition [25]. Therefore, it is imperative to allow people to choose between paper-based *and* digital self-management resource mediums depending on their preferences and circumstances. Further, as the delivery of self-management interventions does not have a typical medium (digital, paper-based, verbal, or flexible delivery), delivery mode (face to face, digital, telephone, or hybrid), or support (self-directed, clinician, peer supported, or blended) [4], offering a suit of flexibly delivered personalized programs may help maximize the reach, acceptability, and appropriateness of self-management interventions when delivered in real-world services.

This study reports the translation of the CORE study self-management resources from the United Kingdom context to the Australian context, including the adaptation of paper-based resources used in the original CORE trial and the development of a digitally based resource guided by the original paper-based tools to provide flexible delivery options for consumers. Following the strong tradition of co-production [2,19], participatory design methodologies were selected for this study to adapt both the paper-based resources and develop the digital self-management resource. Integrating user feedback into the design of digital mental health interventions is the gold standard [26-30] and improves engagement with digital tools for serious mental ill-health [31]. Best practice recommendations emphasize that researchers should publish descriptions of development work, including a description of how design features are influenced by user feedback [32].

### Goals of This Study

The overarching aims of this research study were to (1) translate the CORE paper-based and peer-facilitated self-management resources to the Australian context for successful implementation and (2) co-design and user test a digital prototype of the CORE peer-facilitated self-management resources.

## Methods

### Study Design

**Figure 1:**
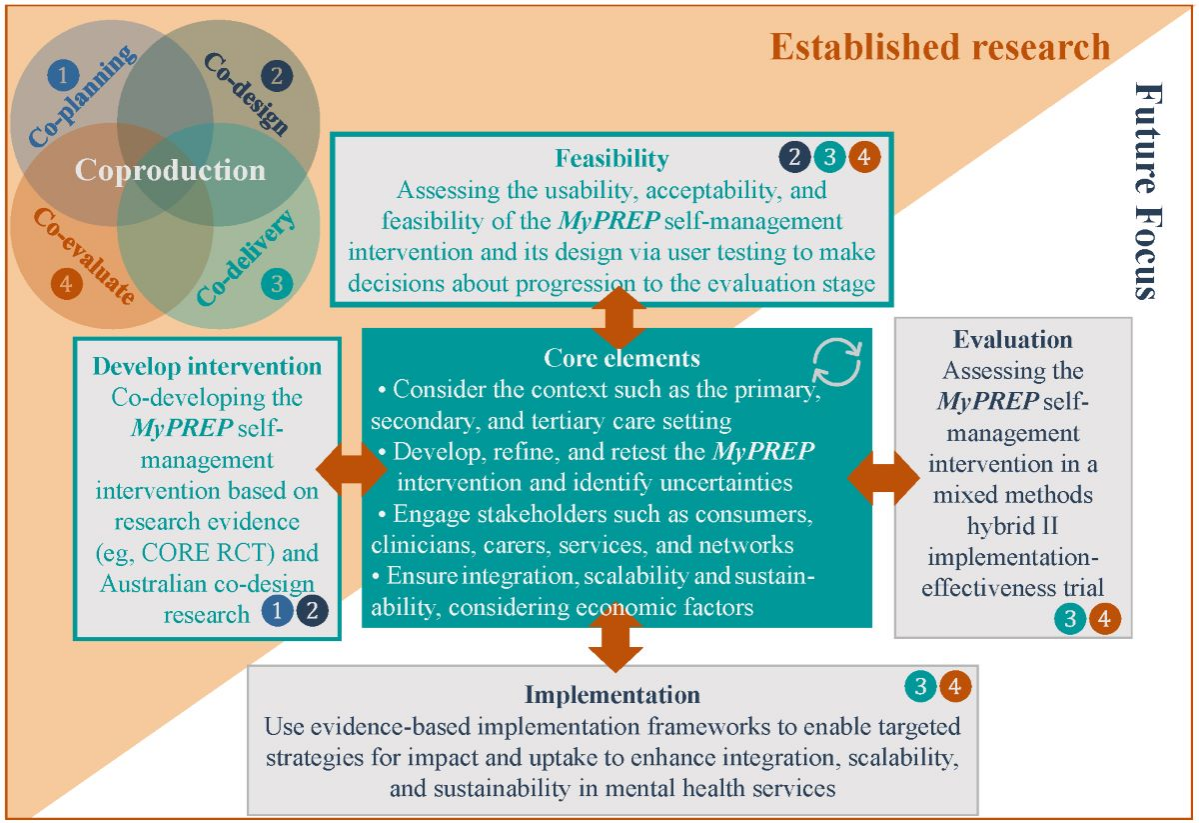
The co-production and Medical Research Council frameworks used in My Personal Recovery Plan (MyPREP) development. CORE: Crisis Resolution Team Optimisation and Relapse Prevention; RCT: randomized controlled trial.

This study adopted a participatory design, user testing, and rapid prototyping methodologies. Two frameworks (Figure 1) provided an evidence-based structure for the co-design process: (1) the 2021 Medical Research Council framework for developing complex interventions [18], including developing, testing the feasibility, evaluating, and implementing the intervention (note that this study focuses on the development and feasibility stages of this framework) and (2) the co-production framework [33], which seeks consumer leadership and input from the outset, including co-planning, co-design, co-delivery, and co-evaluation. In this study, co-planning involved collaborators and researchers with lived experience informing the protocol and study design, ethics application, and recruitment; co-design involved the recruited people with lived experience defining, conceptualizing, evaluating, and designing the prototype; co-delivery involved lived experience researchers facilitating the aforementioned co-design research; and co-evaluation involved the knowledge transition team having representation from people and researchers with lived experience for analyzing and writing up the results.

### Participants

The inclusion criteria for study participation required participants to be aged ≥18 years; self-identify as having a lived experience of using a mental health service, experiencing a mental health condition, or working in a mental health peer support role; have English proficiency; and be able to complete the informed consent processes.

### Co-Design and User Testing Stage 1

The first round of co-design and user testing of the original co-designed UK version of the CORE paper-based self-management resource (which had five sections: (1) *moving on again after a crisis*, which focused on resuming routines and community support; (2) *keeping* well, which focused on activity scheduling and health-promoting behaviors; (3) *managing ups and downs*, which focused on relapse prevention; (4) *goals and dreams*, which provided goal-planning tools; and (5) *making a personal recovery plan*, which provided psychoeducation in a recovery-focused manner) [2,19] was conducted with 6 participants through audio-recorded, one-on-one, and 90-minute user experience sessions, which were held face to face or via a digital video chat depending on participant needs. Interviews were conducted by either a lived experience facilitator or a mental health clinician. During the sessions, facilitators engaged the participants in 3 phases of participatory design processes, namely discovery, evaluation, and prototyping. In the discovery phase, the facilitators used open and prompted discussions to explore participants’ practices, goals, values, and needs in relation to the self-management of serious mental health conditions. In the evaluation phase, participants tested and evaluated the self-management resource, focusing on its strengths and weaknesses. In this phase, the think-aloud methodology [34] was used to gauge the usability and desirability of the design of the paper-based self-management workbook. After this, the facilitator focused on early prototyping with participants, discussing what a digital version might look like to inform the development of a potential digital prototype. Before the end of the first round of co-design and user testing, participants were asked about their basic demographics and their views on the usability of the CORE paper-based resource through an adapted version of the System Usability Scale (SUS) [35].

### Knowledge Translation Stage 1

A knowledge translation team (which included representative stakeholders with lived experience, peer support, clinical, research, and technology backgrounds) was formed and regularly met with the lead researcher (first author) via digital meeting platforms. The knowledge translation team updated the UK version of the CORE paper-based self-management resource to an Australian version based on the stage 1 feedback. Further, the knowledge translation team used the participant co-design and user testing feedback to build a high-fidelity digital (alpha) prototype. To this end, the knowledge translation team engaged in an interactive process of synthesizing, exchanging, and applying knowledge [36]. The ultimate goal was to translate user testing feedback into practice, organizational management, technology development, and policy reform [36]. During stage 2, the tool was renamed My Personal Recovery Plan (MyPREP, which will be used henceforth) by the knowledge translation team.

### User Testing Stage 2

A subsequent round of user testing of the high-fidelity digital prototype took place with 4 new participants using the think-aloud methodology to gauge the usability and desirability of the design of the Australian paper-based self-management workbook. After the tool was adapted based on the identified problems and suggestions, a group of 6 PSWs who would be piloting the tool in their community mental health service were provided with access to the digital and paper-based tools for a final round of qualitative feedback.

### Data Analysis

The audio-recorded sessions were transcribed and anonymized. The qualitative data were subsequently interpreted using a previously established knowledge translation process for participatory design studies [37]. Specifically, the knowledge translation team developed a coding framework outlining all key concepts. Data were coded in the NVivo software (version 12; QSR International) using this framework. Data interpretation followed established thematic techniques [38], which involved an iterative and reflexive process of reading, coding, exploring the pattern and content of coded data; reflection; and discussion. Similarities and differences in opinions were examined, and differences were dealt with through discussion to reach a consensus. The knowledge translation team also identified themes and key learnings to inform the customization and configuration of the paper-based MyPREP program and the digital high-fidelity prototype. Acceptability scores were calculated using the standard SUS process [35]. Frequency and descriptive analyses of the quantitative data generated in user acceptance–testing sessions were conducted in SPSS (IBM Corp).

### Ethical Considerations

Ethical approval for this study was obtained from the University of Sydney’s Human Research Ethics Committee (HREC reference number 2019/571). Informed written consent was obtained in advance from all the participants in the qualitative sessions of this study. We assert that all the procedures contributing to this work comply with the ethical standards of the relevant national and institutional committees on human experimentation and with the Helsinki Declaration of 1975, as revised in 2008.

## Results

### Participant Demographics

All 10 participants in the first 2 rounds of user testing identified as having lived experiences of a mental health condition and mental health service use, with 3 (30%) of the 10 participants identifying as male, 3 (30%) residing in regional areas of Australia, and 6 (60%) having a job as a PSW. The final protype was then appraised by 6 (male: n=3, 50%; female: n=3, 50%) additional PSWs, all working in urban and suburban areas of Sydney, New South Wales, Australia.

### Main Themes

The key themes identified throughout the co-design and user testing sessions were related to (1) the need for self-management tools to be flexible and well-integrated into the mental health services used by participants, (2) the importance of language and how language preferences vary among individuals, (3) the need for self-management interventions to have the option of being supported when delivered in services, and (4) the potential of digitization to allow for a greater customization of self-management tools and the development of features based on individuals’ unique preferences and needs. Further, summaries of the full recommendations of adaptations to the MyPREP paper-based and digital prototypes are presented in Multimedia Appendix 1.

#### The Need for Integrated and Flexible Self-Management Tools

Most participants confirmed that there was a lack of routine provision of self-management tools supporting recovery in community mental services, and these tools should be offered as a standard as early as possible:

Honestly, I think it might have been really beneficial to start [using self-management tools] while I was inside [the hospital]. You know that that way I’m not sort of just being discharged into sudden loss of support. I’m being discharged with a plan I have got a set of actions and a structure to go back to.

P3, user testing round 1

These participants expressed that there was a strong need for the flexible delivery of self-management tools in mental health services. That is, services should not provide just one type of medium to deliver interventions, as the needs of individuals are not uniform:

Individuals may experience barriers [to using self-management tools] due to their lack of use in technology. Or finding it challenging to adapt to technology this resulting in becoming fearful of using technology.

P4, user testing round 2

From what I’ve observed, especially if it’s like 60 and above, they love paper versions, even though like, I mean, some are tech savvy. Yeah, they do feel that comfort in what they know as well. But I guess it’s just about educating them a little bit more about the technology and allowing them to adapt as well. So having both options is always, always better than not having an option.

P4, user testing round 2

Self-management tools also need to be well integrated with other plans and documentation offered in services, and digital platforms could enable this integration:

We get given a lot of stuff when we’re coming out of the crisis, particularly for going to hospital discharge. You’ve got a discharge plan, you’ve got all your appointments, you got your medication sheets. You may even have a safety plan depending on which program you’ve gone through. So under a suicide attempt or you, we thought there might be a safety plan that’s developed as well. So I think we just need to consider how this works in conjunction with all the other bits and pieces that might be provided.

P4, user testing round 1

#### Language

A major theme identified in the co-design and user testing sessions was language. Some words need to be changed to reflect the common language in Australia compared with the United Kingdom. People preferred less formal and less clinical language. Participants also liked that the language used generally did not make assumptions about the person’s situation:

I like that it’s like everyday language ‘the things I need to sort out.’ Yeah, I like that.

P1, user testing round 1

[When speaking about the ‘people and places I can turn to’ page] I really like it. And what I like about it is it’s not assuming that those people are family members. You you’re not making people feel worse by, you know, having to put down friends or colleagues or neighbors or whatever, because family is not in your life. So ‘what support would I like?’ Yeah, that's great. Nice basic phrasing there.

P4, user testing round 1

The participants also discussed the importance of not having too much text on each page, as it can become overwhelming and distracting for users, especially as impaired cognition and concentration can be symptoms of mental health conditions. Decreasing the number of words on the screen should be a priority for increasing usability. This could be done by incorporating images, especially infographics, and additional features, such as read-out-loud audio and avatars:

Language, is really, really important. The less that’s on a screen or on a paper or on a document is better. Yeah, because you can’t take it in. It’s too much noise. It’s too busy. And if my concentration is already impaired, it’s not going to help me stay engaged. I’m just, I just won’t get engaged.

P2, user testing round 2

One of the participants said the following about the name of the tool:

I don’t like the words. Actually, at first I don’t like the word recovery, I don’t like the personal, and I certainly don’t like plan... [the workbook needs] something more casual ...it’s the balance between not being too positive that can be invalidating too.

P1, user testing round 1

Further, participants emphasized that the language used in self-management tools needs to be empowering, promote self-agency, and be sensitive to the mental health situations of people, as mental health can fluctuate. Although it was generally agreed the language was used in MyPREP met this need, there were few words identified by some participants as having the potential to be disliked by some users of the tool, such as “recovery,” “triggers,” “moving on after a crisis,” “plans,” and “goals”:

And now going back to the word recovery, if we’re going to be changing it in certain instances, personal recovery. And it’s interesting because now we are having this challenge around the word recovery. Yeah, some of us are now saying, hang on, many of us are not recovering from anything. If we want to look after health and wellbeing proactively, yeah. ...where are you on your journey? Exploring your health journey? Or your life journey, whatever it is that you want to use.

P2, user testing round 2

Views on language were mixed, with some reporting that they liked the language that others did not: “oh I like the words ‘moving on’” (P1, user testing round 1). Moreover, some participants reported that some terms should remain in the tool. For example, some felt that the term recovery should remain, as it promoted autonomy:

You know, I think recovery is like very self-empowering. And that puts a lot of independence on people. ...And so just thinking about perhaps placing the autonomy a little bit more on the individual as well. So that is self-directed, that is more self-directed yeah.

P1, user testing round 1

Working on the tool with support (particularly from a peer worker) would enable these conversations about language to occur.

#### Supported Facilitation

Self-management tools need to be customized and personalized to the individual, and many participants felt that this needs to be carried out through conversations with facilitators and supporters, such as PSWs:

Yeah, I think, um, this [using MyPREP] would be excellent to do again, through a conversation. Yes like where, if the person accessing the service would be talking with a peer worker and then, so, yeah, ‘I believe this is what recovery is,’ ‘what you think recovery is about,’ ‘what is recovery for you,’ that sort of thing.

P2, user testing round 1

These conversations “made it [the paper-based self-management workbook] so much more human” (P1, user testing round 1), especially because facilitators such as peer workers can tailor conversations based on individual needs while using the tool as a guide:

Yeah, I would tailor it. So it’s. Yeah, that is more for a conversation. Yeah, where the peer worker could be like, ‘So what are some signs that I could look for in you that might make me think you're not going so well?’

P2, user testing round 1

Further, the accompanying psychoeducation component that helped contextualize the workbook was located at the end of the booklet in the original UK resource. Participants highlighted that this needed to be integrated with the MyPREP module activities (note that this was actioned for both the digital and paper-based Australian versions of MyPREP). Moreover, it was emphasized that the content of this psychoeducation component could be discussed with someone such as a peer worker, clinician, or supportive other:

I think that [psychoeducation] needs to be at the front [of the MyPREP tool]. Yeah, the way I would work with it is in that context, I’d be looking through it and go, oh okay, I’ll go to the next page and then I’ve probably filled in a lot of that stuff before I’ve got to the end. It is where all the guidance around this. ...It might not be read, so just all of this content could be put it in within the chapters. Yeah, that or a statement at the front of the elements that you feel from the information on how to help you fill it in was confined to the back of the book. A plain guide just say, Look, don’t just launch into it. But there’s some further guidance and support there on the back of the book that you can work with a friend or someone through it all with your worker. You know, because that is a lot of text there, and we are assuming the people have the ability to read and comprehend all that information.

P4, user testing round 1

#### Additional Desirable Features and Functions When Digitized

**Figure 2:**
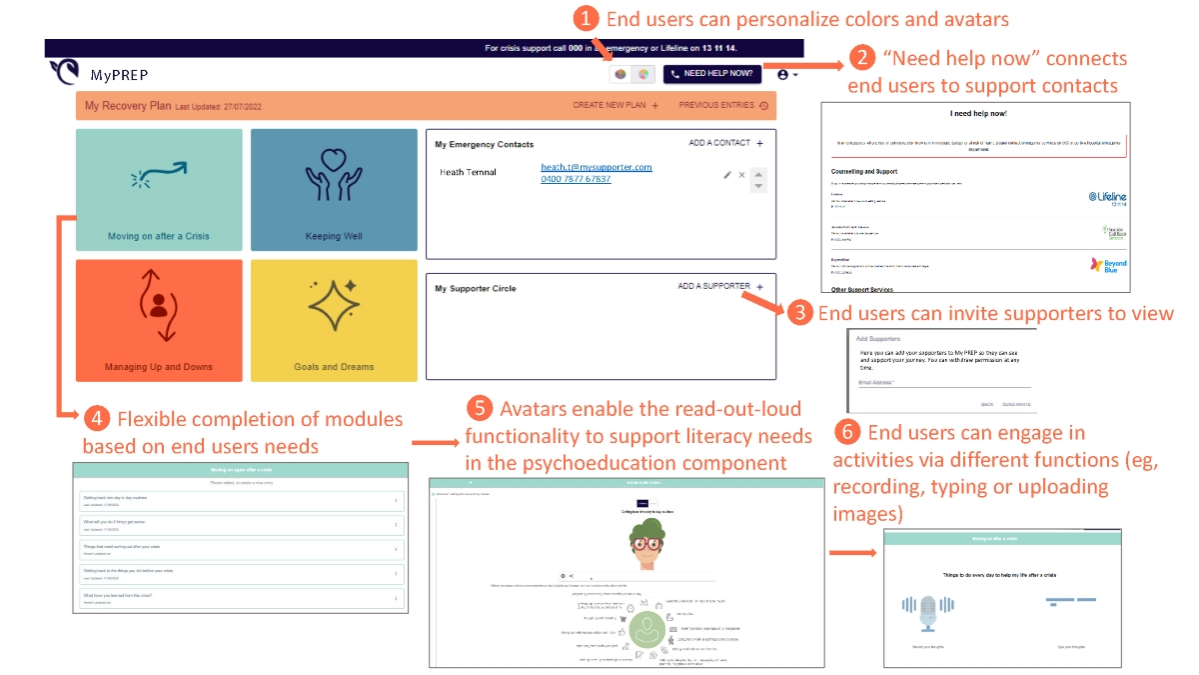
Exemplar additional features and functions. MyPREP: My Personal Recovery Plan.

Additional features and functions beyond those of the paper-based MyPREP workbook were highlighted by participants during the co-design process. Exemplar additional features and functions are displayed in Figure 2 and described in detail below (refer to Multimedia Appendix 2 for enhanced visibility of features on each page).

On the basis of the feedback gathered during the user testing sessions, *customization* by the users themselves was enabled in the digital version so that they could adapt the content to their preferences. End users could make changes by configuring the color pallet and the voice and image of their avatar and were able to alter and flexibly select the MyPREP modules. The ability of end users to customize MyPREP was seen as very important, as it enabled a feeling of ownership over the tool. For example, the color pallet could be changed to reflect not only the preference but also the mood of the participant, which was seen as important in mental health:

I think it’s like there’s dark colors and light colors pastel. Yeah, I like that. I think that’s really nice because it’s like I said, you’re going towards personal, like being personal. And this gives choice of being personal as well. It’s cool.

P4, user testing round 2

When you are unwell, you only see fog, color can break through.

P1, user testing round 2

The customization of the image and voice of the avatars in the program was seen as trauma informed (as it had the potential to decrease inadvertent traumatization if someone had a previous traumatic experience with a person of a particular sex) and gender inclusive (as voice and image could be changed) and helped tailor the avatar according to the individuals’ backgrounds, such as their age. This was seen as an important feature, and additional languages could be easily integrated into the MyPREP package in the future:

You just have to think of the LGBTQ plus community as well... I think that’s because if you do like a said male or female, it’s just like ‘why only two?’ Yeah, you know, but then if it’s like I said a plant [an alternative avatar on MyPREP], it’s very cute and it has a smiley face. And I really like that because I think especially in the new generation, if you think about it, a lot of like animations are popping up as well. So, if you want to adapt to the younger generation, they might really enjoy this thoroughly.

P4, user testing round 2

A new feature, which was not possible in the paper version but enabled through digitization, was a “need help now” button, which was suggested by participants and provides the contact details of crisis support and additional mental health support services. This feature was developed after participants in the user testing sessions recognized and acknowledged that creating a MyPREP plan could potentially be confronting, and the individual may want additional support from others to process the information or additional mental health crisis support:

If I’m not in a good space and I’m trying to read it, I want to know I’ve got a safety button. I can go to hey, look, I’m feeling like shit, OK, and I might be reading this and you know what? I actually want to talk to someone, or I want to write to someone or chat to someone. Yeah, and I get blocked. Yes. I think, you need to have a safety, safety spot, do you need help now? Something. The hope is you read it, but it’s also a risk thing that, yeah, I think you need this.

P2, user testing round 2

Two other new features were “my emergency contacts” and “supporter circle.” The emergency contacts feature allows users to enter the contact details of people and services they can contact during a mental health crisis. In addition, the supporter circle function was designed to allow users to share their recovery plan entries with people in their support circle (such as carers, health professionals, and peer workers) so that they could view their MyPREP entries. One of the participants expressed that it was important that the user be prompted to input contacts for both features when signing in for the first time to provide a safety net if the user became distressed in the process of completing the plan and suggested displaying these important contacts on the dashboard at all times to provide a sense of support to users:

OK, I would want them to have their emergency contacts sorted and their support circle sorted because they can’t move on after a crisis. If they don’t, if they’re unaware of their support circle, then we can’t help them to do that either if we don’t know. Especially and then managing up and ups and downs like they’re going to need their support circle and their emergency contacts.

P3, user testing round 2

All participants acknowledged the usefulness of the supporter circle feature in providing access to the user’s professional support network, including psychologists and support workers, if the user wanted their MyPREP entries to be shared:

People that are supporting you that you want to know what’s going on? Mm-Hmm. Yeah. So. I mean, you can have them invited by, or you can share a copy, so I mean, that would be really useful for clinicians and obviously peer workers and mental health workers...

P3, user testing round 2

It was emphasized that some may not use this feature because of privacy concerns, particularly with family or carers: “some people really don’t like family seeing this stuff” (P3, user testing round 2). Importantly, permission for viewing access could also be withdrawn by the end user at any stage:

The major function of the digital self-management tool is to assist users in systematically mapping and recording personalized self-management strategies in response to prompts to assist in their recovery from a mental health crisis. A feature that facilitated this was the avatar for read-out-loud functionality, which was viewed as especially important for those who held a preference for this or who had lower literacy skills. Further, each activity could be completed in a variety of ways based on the individual’s needs. Specifically, end users could engage in MyPREP activities by uploading voice recordings, images, emojis, and text. This was seen as a highly inclusive feature:

Oh, we got little icons. I would probably put a smiley face. Awesome, and then you can upload stuff. That’s cool, that’s like really personalized. Awesome! ...I think people get really anxious when they feel like they’re being recorded or they don’t like hearing their voice playing back, I know I don’t. So I would always go for the text. ...I think some people might like it. Some people might not want to type.

P3, user testing round 2

These features were also seen as important motivating factors that promote better end user engagement with MyPREP:

I don’t know if people are going to struggle with doing these entries, because if they might think, OK, what is the purpose of it? But if it’s more fun and personal, it’s more fun. So, it’s like okay, I’m going to do this one. I might even put a photo of me in it and get that done. But if it’s always just bland, I don’t know. People might be like, well, especially, you know, you want that motivation. But I think this is really cool.

P4, user testing round 2

The MyPREP module activity entries made by the end user were also editable, and there was a function in which entries could be saved so that the end user could keep a record of the changes:

This is really awesome. And it will keep the track as well. You can kind of say you can kind of go back, it can be like okay I was feeling not great on this day, what happened that day as well? ...Yes. So, you can kind of see that progress, especially for consumers who do enjoy that progress.

P4, user testing round 2

### The SUS

At the end of each user testing session, participants completed the SUS. The mean and range are listed in Table 1. The MyPREP paper version received a total SUS score of 71, indicating C+ or “good” usability. The digital version received a total SUS score of 85.63, indicating A or “excellent” usability.

**Table 1:** System Usability Scale (SUS) usability scores for the paper-based and digital versions of My Personal Recovery Plan (MyPREP)^a^.

SUS items	MyPREP paper-based version, mean (SD; range)^b^	MyPREP digital version, mean (SD; range)^c^
1. I think that I would use the Personal Recovery Plan frequently.	2.4 (1.3; 0-3)	3.25 (0.5; 3-4)
2. I found the Personal Recovery Plan unnecessarily complex^d^.	2.4 (1.3; 0-3)	3.75 (0.5; 3-4)
3. I think the Personal Recovery Plan would be easy to use.	3.0 (2.1; 0-5)	3.5 (0.6; 3-4)
4. I think I would need the support of a support person (e.g., a peer support worker) to use the Personal Recovery Plan^d^.	2.2 (1.5; 0-4)	2.75 (1.0; 2-4)
5. I felt the various sections in the Personal Recovery Plan were well integrated.	3.6 (0.5; 3-4)	3.5 (0.6; 3-4)
6. I thought there was too much inconsistency in the Personal Recovery Plan^d^.	3.2 (1.3; 1-4)	3.5 (1.0; 2-4)
7. I would imagine that most people would be able to use the Personal Recovery Plan easily.	2.2 (1.3; 0-3)	3.0 (0.8; 2-4)
8. I found the Personal Recovery Plan very cumbersome^d^.	2.8 (1.1; 1-4)	3.5 (1.0; 2-4)
9. I would need to learn a lot of things before I could start using the Personal Recovery Plan^d^.	3.2 (1.3; 1-4)	3.25 (1.0; 2-4)
10. I would feel very confident using the Personal Recovery Plan myself.	3.6 (0.8; 2-4)	3.75 (0.5; 3-4)

^a^The total SUS scores for the paper-based and digital versions were 71.5 and 84.4, respectively.

^b^Of the 6 participants in the first round of user testing, 5 (83%) completed the SUS for the paper-based version.

^c^Of the 4 participants in the second round of user testing, all 4 (100%) completed the SUS for the digital version.

^d^Reverse scored item.

In a final meeting before endorsing MyPREP for piloting, the researchers met with 6 PSWs, and the tool was presented and discussed; these peer workers were then given access to the MyPREP intervention to gather their feedback. At this meeting and at follow-up, only positive additional feedback was relayed, and the peer workers emphasized that they were excited to use the tool in their service:

I have reviewed the Workbook and honestly couldn’t find much wrong with it at all!! It’s great and I am very excited to put it into practice. My team had no feedback to give on the digital version other then it looks great and can’t wait to see it used with clients in practice!!

Peer worker 1

## Discussion

### Principal Findings

In this study, we present the user testing of an Australian version of the paper-based MyPREP and the co-design and user testing of a digital version of MyPREP. The key themes identified throughout the co-design and user testing sessions were related to (1) the need for self-management tools to be flexible and well-integrated into mental health services, (2) the importance of language and how language preferences vary among individuals, (3) the need for self-management interventions to have the option of being supported when delivered in services, and (4) the potential of digitization to allow for a greater customization of self-management tools and the development of features based on individuals’ unique preferences and needs. The resulting tool was subsequently rated using the SUS, which indicated that the end users involved in this study rated the paper-based tool as “good” and the digital version as “excellent.” Taken together, these results suggest that the digital prototype has valuable potential for use in mental health services and support the progression to piloting MyPREP in mental health services to inform a future large-scale randomized controlled trial (RCT). Early studies providing detailed descriptions of intervention development, such as this study, are now called for as best practice [32]. Specifically, it is important to include details such as important decision-making steps across all stages of development, explanations of how the information gathered from intended end users was carried out, and how intended end user input influenced how design features were incorporated into the design.

### The Need for Implementation Research

The participants in our research also highlighted the need for the integration and coordination of self-management interventions and tools within services, as there can be a multitude of staff, documents, and plans (such as suicide prevention plans, discharge plans, wellness plans, and medication adherence plans) involved at various points throughout a consumer’s or a service user’s journey in a mental health service. Further, although the digital version of MyPREP was seen as being able to be used independently by end users at their home or within health settings, participants emphasized the need for such tools to be delivered with support from others, particularly PSWs. Although this may be explained by the fact that many of the participants were peer workers or consumer advocates themselves, this finding mirrors the current movement within mental health services toward the holistic inclusion of lived experience expertise [39]. Further, integrated staffing models may also be associated with better recovery, including better social functioning [40]. Taken together, implementation-focused research is needed to determine how MyPREP is actually delivered and how it can be optimized once it is introduced in real-world settings.

A recent systematic review of supported self-management interventions for people with serious mental health conditions found that there is a current lack of studies focused on implementation and that even fewer studies are based on implementation science theories [8], which are summarized in the taxonomy of implementation outcomes [41]. Although RCTs remain the gold standard approach to informing clinical decision-making and drawing causal inferences [42,43], there remain unacceptable research-to-practice gaps and a disconnect in generalizability and performance between an extremely controlled clinical trial environment and highly complex real-world mental health environments [44]. Implementation science, which is the formal study of methods to promote the systematic uptake of research findings and other evidence-based practices into routine practice, can bridge the clinical evidence-to-practice gaps by improving the quality and effectiveness of health services [44]. The hybrid implementation-effectiveness design is an innovative and rapid solution to provide high-quality evidence on effectiveness and implementation simultaneously [44,45], making it the ideal next stage of the research pipeline for the MyPREP project to determine effectiveness and implementation. This step is particularly important in our case, as the basic content of MyPREP has already been tested successfully in the CORE trial in the United Kingdom [16].

### Accessibility of MyPREP

The digitization of MyPREP may have allowed it to become more accessible to end users when compared with the paper-based version, and this may partly explain the increase in usability scores, as measured using the SUS. For example, the use of the read-out-loud feature via avatars was viewed very positively by participants, as was the provision of options for writing, recording audio, and uploading images when completing MyPREP module activities. A recent systematic review suggested that developers of self-management interventions should adapt interventions to ensure greater inclusivity for participants with less formal education, as it was found that educational level was associated with engagement [8]. This is particularly critical for individuals with severe mental health conditions, given the increased rate of lower education in this cohort [46]. Indeed, the participants in our study repeatedly emphasized that plain and informal language was preferred. Further, we used Design for Dignity Principles, Web Content Accessibility Guidelines, and International Organization for Standardization standards for process improvement, safety, and quality (eg, 9241-11 ISO standard) to promote digital accessibility and ensure that the features are usable and acceptable for people with accessibility issues.

### Customization of MyPREP

Customization embedded within MyPREP, such as the ability to change the color pallet and choose an avatar, was a clear theme throughout the co-design process and was rated very positively in the user testing sessions of the digital tool. Customization was important, as MyPREP is a self-management tool; the participants emphasized that these self-directed changes to MyPREP fostered a sense of autonomy, control, choice, and ownership, and this should be embedded as a standard in all features. Reviews in this area recommend that the content of self-management interventions should be tailored to the service users and have the flexibility to be personalized and customized, especially as interventions were found to not always fit end users’ needs [8].

In the future, there are plans for MyPREP to be customized further. A major example is enabling control over language. Specifically, feedback concerning the language used for the names of modules (eg, “my recovery plan,” “moving on after a crisis,” and “goals and dreams”) was liked by some participants in the user testing sessions, but not by others. This was despite MyPREP being co-produced and going through iterative co-design cycles with people with lived experience of serious mental ill-health [2,19]. The solution to this lies in customization, through which end users can adapt the names of the modules to suit their own set of beliefs and even remove modules that are not relevant to them from their MyPREP dashboard.

### Limitations

This is a preliminary iterative co-design and user testing study and should be viewed as such. Similar to most user testing studies, our sample of service users was small (eg, the study by van der Krieke et al [47]). Further, we used not only advertisements across networks and services but also snowball sampling, which is a type of convenience sampling. A major disadvantage of such convenience sampling is that it risks a nonrepresentative study sample. In our case, the study sample was quite diverse in terms of age and sex. However, a large proportion of participants were consumer advocates and peer workers with considerable mental health knowledge and expertise. Further, those recruited for this study might have had a particular interest in working with digital health tools, which may have introduced avidity bias and may explain the very high SUS acceptability scores for the digital version of MyPREP. The next stage is piloting MyPREP in services to increase the representativeness of our sample and make iterative adaptations to MyPREP based on user feedback. However, overall, as the original MyPREP was trialed in the United Kingdom with at least 275 crisis care service users in a large-scale RCT (with 441 service users enrolled in the trial), this may suggest that the representativeness of our sample at this point does not pose a concern for the next stage of piloting of the Australian version in real-world mental health services.

Another limitation that is common in user testing (eg, the study by van der Krieke et al [47]) is that the presence of the facilitators over a digital meeting platform during the testing sessions may have affected the views of the participants, as they might have felt reluctant to be critical. We do not expect this to be a major limitation; however, throughout user testing, the facilitators continuously emphasized that this was an opportunity to improve the paper-based and digital prototypes and encouraged discussion around problems and areas for improvement.

### Conclusions

The co-production of the MyPREP self-management intervention and associated research in Australia are currently in their early stages. However, this co-design and user testing phase is a crucial step in adapting MyPREP to the Australian mental health setting and digital context. However, the current findings may remain relevant to implementation in any setting. Overall, the co-production process is vital, as service-wide implementations that fail to consider end user needs and organizational structures often encounter problems. Indeed, Killikelly and colleagues [31] suggest that both co-design and support from mental health staff or researchers when using the tool are 2 features that are associated with successful implementation and improve engagement with digital tools for people experiencing serious mental health conditions. To avoid implementation issues, it is essential to involve consumers and peer workers, who may support the delivery of MyPREP from the outset. In this study, feedback from these end users highlighted a strong desire for the personalized delivery of self-management interventions that offer choices and options, considering individual end users’ different needs and circumstances. MyPREP has worked toward addressing this need by offering digital and paper-based mediums, providing options for how delivery is supported (ie individuals can choose their supporters), increasing accessibility (eg, avatars and voice-recording options), and allowing customization (eg, customization of the color pallet and choice of avatars) based on users’ preferences and needs. To strengthen MyPREP's implementation in real-world Australian mental health service settings, implementation-effectiveness piloting and robust trialing are required to test and refine the tool.

### Lived Experience Commentary

In our view, co-production is an umbrella term for co-family, co-creation, co-planning, co-implementation, and co-evaluation. This is a concept and philosophy in which collaboration with lived experiences is paramount. It is a way to cocreate new interventions, improve systems, and solve problems and is now being adopted in many public health policy arenas, including research.

This has moved far beyond “consumer participation,” which was enshrined in 1992 in the Australian mental health policy (1992 National Mental Health Policy endorsed by Australian Health Ministers). Back then, this was ad hoc and tokenistic. Now, as “co” is becoming more widespread, there are more genuine attempts to learn to integrate co-production as a way of doing research across the board.

In research, with co-production, we are witnessing a move from consumers and carers (or those with lived and living experience) voices from being “subjects of” to being equal collaborators, who work with researchers to influence change that benefits the service and system user. This research project has done just that. By having lived experience experts lead, participate in, and contribute to the design and facilitation of the research, we actively used lived experience knowledge and expertise in recovery planning through various stages. The recruitment of participants, data collection, workshops, one-on-one interview formats, and questions were developed and led by lived experience.

In this project, a trusting respectful alliance between research and lived experience evolved organically and naturally, providing a solid foundation to “do” this project with passion and enthusiasm, creating a safe and supportive atmosphere emulated in dealings with others. The interviewees felt more than comfortable to offer their time generously, sharing their insights into and thoughts on what would work well and what should be improved to increase engagement with and the use of this recovery-based intervention.

During different phases of this project, reciprocal positives emerged for the research team. Empowering and enabling each member to consider different perspectives and interpretations. Embracing new ways of thinking created a richer understanding of issues not considered previously. This was articulated in the ways in which the research was focused and conducted. On reflection, lessons on how this research approach will inform similar ongoing collaborations should be considered, where research projects embrace and welcome different skill sets, experiences, and knowledge.

## Appendix

Multimedia Appendix 1 User-testing reports.

Multimedia Appendix 2 Example additional features and functions.

## Abbreviations

CORE: Crisis Resolution Team Optimisation and Relapse Prevention
HREC: Human Research Ethics Committee
MyPREP: My Personal Recovery Plan
NICE: National Institute for Health and Care Excellence
PSW: Peer Support Worker
RCT: randomized controlled trial
SUS: System Usability Scale

## Data Availability

The qualitative data sets generated and analyzed during this study are not publicly available, as they are lengthy interview transcripts from which quotations used in this publication were selected, to ensure that individual services or participants cannot be identified. The data sets are available from the corresponding author upon reasonable request.
