# Supplementary material for: Co-Production of a Flexibly Delivered Relapse Prevention Tool to Support the Self-Management of Long-Term Mental Health Conditions: Co-Design and User Testing Study
Source: JMIR Form Res. 2024 Feb 23;8:e49110. doi: 10.2196/49110 (PMC10926903; doi:10.2196/49110)
Supplement: Multimedia Appendix 1 [file formative_v8i1e49110_app1.pdf]

User Testing Report Phase 1

My Personal Recovery Plan – Paper Based Version

Overall Recommendations

- **Design**
    - Use the current colours and images
    - Increase size of font of title pages
    - Use bold/colour to better emphasis key words
    - Reformat some pages for better readability

- **Language**
    - Potentially change the title of the workbook
    - Some words need to be changed to reflect common language in Australia
    - Preference for more sensitive/ stigma reducing language
    - Preference for use of personal pronouns

- **Content**
    - Add more infographics to reduce the text
    - Remove or generalise religious matter
    - Add examples/prompts for some of the exercises
    - Utilise the psycho-education section at the back and move throughout the document without overwhelming user with too much text

Summary of participant findings for each page of the paper-based workbook

**Page #**	**ID #**	**Findings**	**Notable Findings**
Cover	1	Tortology Dislikes - name of plan (unsure or recovery and plan), suggests “Contributing lives, thriving communities” Likes - design (clean, simple, not overwhelming).	- The name of the plan did not resonate with everyone. 50% participants liked ‘Personal’ and 50% participants felt ‘recovery’ was not the accurate/best choice.

	2	Likes name of plan though mentions recovery is not always linear so suggests journey. Booklet is a bit dull and could be more engaging. Tree image - could be misleading, suggests an image that is applicable to everyone.	Importantly, ‘recovery’ is not used by Indigenous groups.<br>- Most liked the design, especially the image of the plant.
	3	Likes ‘personal’ in the name of the plan. Likes plant image.	
	4	Likes the use of ‘personal’ in the name of the plan - highlighted and subtle. Notes the use of boxes can be overwhelming for a consumerLikes the plant image and denotes growth and inspires hope.	
	5	Dislikes ‘recovery’ in the name of the plan. Notes ‘recovery’ is not a word used by Indigenous groups and its word used in a clinic setting.. Suggests ‘Wellness plan’. Likes the design - look and feel, font, simple, room to write, big text. Consider adaptations for different groups/languages. Digitalized give 3 or 4 options - in terms of adaptations to culture, language and/or design?.	
	6	Suggests the title should be in bigger font for better readability. Suggests ‘Personal’ should be a darker shade of green to match the tree. Notes, hopes it's a growing tree, mentions trunk - to include. Also, there’s possibly too much white space and should be toned down.<br><br>Re: whole booklet<br>- Likes the boxes, though notes consumers could run out of space to write. Need to find a balance between lines and blank space.<br>- Suggest including prompts, use tick boxes and drawing to help consumers to express themselves who are less verbal.	
Credits	1	-	
	2	-	

	3	-	
	4	Agrees - ok	
	5	Good to know the source of the information.	
	6	-	
Intro	1	To me recovery means - unsure of recovery could be mistaken for medical	- 33% participants commented on the word ‘recovery’<br>- word could be mistaken for a medical term<br>- consumer may not readily understand the meaning of recovery
	2	-	
	3	Suggests including ‘for me’ and ‘my’ in the intro statement.	
	4	Notes ‘what understanding does the consumer have of recovery? Has taken them years to come to an understanding. consumers may feel intimidated by the exercise if completing it on their own. Suggests complete with someone/consultation, include an example, beneficial to complete whilst still in care before discharge, in fact helpful.	
	5	Suggests more room to write responses. Notes, the booklet hasn’t yet asked about them - Who am I? Who is important in my life? Where are we heading?	
	6	Notes, image detracts from the content.	
My space	1	Likes the idea of the page - My space.	- 50% participants suggested that an explanation and/or examples should be included to give the consumers guidance on the intended purpose of the ‘My space’
	2	-	
	3	Suggests ‘My reflections’ or ‘My space for reflection’.	

	4	Suggests 'My space' section should be more specific and include an example. Using as an art piece, a way to have a consultation.	section.
	5	The page doesn't seem to be in a logical spot. Suggests including an explanation of the purpose of the 'My space' section with examples. Notes the blank page could provoke anxiety in people.	
	6	Agrees - okay	
Contents	1	Suggest change the word "Goal". Like colours.	- 50% participants thought the exercise titles should be reworded.
	2	Dislikes 'crisis'. Suggests adding 'after being unwell' after 'Moving on again after'.	
	3	Suggests 'first steps after a crisis' instead of 'Moving on'. Believes 'moving on' is stigmatizing. Notes many consumers are told to 'move on'.	
	4	Suggests 'Managing a crisis' instead of 'Moving on again after a crisis'. Specifically dislikes the use of 'again' as they feel one is always in crisis. Suggests specifying 'Blank space' is the 'My space' section. Suggests adding text to use the space as another way to express themselves.	
	5	Likes the very simple wording/plain language. Likes the design - colours being very neutral and not bright. Suggests moving the 'Blank space…' statement to My Space section.	
	6	Agrees with wording. Likes the design - blue and purple colours are soft and calming. Questions the use of olive green.	
My space	1	-	
	2	-	
	3	Suggests 'My reflections' or 'My space for reflection'.	

	4	Suggests including an example and make the example specific to the 'My space' section of the booklet.	
	5	-	
	6	-	
7	1	Likes colour.	- 33% participants like the colour of the page.
	2	Suggests omitting 'after a crisis'.	
	3	Dislikes 'Moving on'. Likes feel and colour of design.	
	4	-	
	5	-	
	6	Suggests increasing size of text and moving ⅓ of the way down. Finds the colour of the page to be gentle.	
8	1	Finds the page overwhelming. Suggests providing examples to draw from such as Lifeline, GP and friends/family. Suggests 'Best contact' instead of telephone.	- 33% participants suggest providing examples to prompt answers
	2	Askinging a consumer to list 'people and places I can turn to' could make them feel isolated if they don't have any to list. Suggests moving page to later in the booklet to the section on what I can do.	
	3	Suggests providing a hint i.e. examples	
	4	-	
	5	Likes that the basic phrasing and that it doesn't assume the consumer will list family members. Suggests including FB name and email in addition to phone. Suggest to increase the space for the number of contacts and to	

		make it easy to print off and put on pages as well as provide 7 copies at the end of the booklet.	
	6	Notes, the page is very important, nothing really is missing. Suggests that the ‘What support I would like' box be split into two - 1) who is the person (e.g. psychiatrist, mother) and 2) support desired. Suggests including a prompt below ‘In an emergency I can call’ - local and national support services.	
9	1	Finds the word ‘every day’ overwhelming. Suggests starting with the second exercise, emphasized the use of ‘some days’. ‘Avoid’ may be too strong.	- 50% participants thought the exercise titles should be reworded.
	2	-	
	3	Suggests rewording ‘Things to do every day…’ with something along the lines of what's good.	
	4	-	
	5	‘Not too much pressure, slow down’ - is that stating that this needs to be done? Likes language. ‘Combination to work with peer - sharing helpful stories - is that suggesting to work with a peer support worker or actually discuss with peers?	
	6	Re-organise the exercises to be in vertical order to increase flow of thought. The green text is getting lost. Suggest ‘Things which help me on...’ instead of ‘Things to do…” Suggests to play with wordint, as the text is wordy.	
10	1	Suggests changing the word ‘worse’. Suggests ‘What will I do?’ instead of ‘The action I will take’.	- 33% participants thought the exercise title should be reworded.<br>- 33% like the page as it is.
	2	-	
	3	Suggests ‘Signs that I may be starting to feel worse’ should be reworded to be more specific and to include questions to prompt the consumer to	

		consider. Suggests that the prompt 'The action I will take' includes examples.	
	4	-	
	5	Likes the page - considering the consumer is in control.	
	6	Likes the page -"Ifine"	
11	1	Disliked the page presumably because it feels intrusive. Suggests making the exercises less intrusive. Suggests 'People I need to thank', instead of want.	- The exercise 'People I need to thank' was contentious - 50% participants thought it was intrusive, presumptuous or too early in the consumer's journey to contemplate.
	2	-	
	3	Agrees - ok	
	4	-	
	5	Questions if consumers 'People I need to thank' will understand this point so early in their journey? Suggest the exercise be later in the booklet.	
	6	Confused by what the page is asking the consumer to do. Suggests omitting 'People I need to thank' as they found it to be presumptuous. Suggests changing the box to a vertical order instead of horizontal to help flow and thinking. Suggests 'Problems I need to sort out' needs to be reworded. 4 boxes to help flow and thinking. Choice - multiple things to do.	
12	1	Loves idea of the page - Activities and responsibilities I value. Suggests changing 'responsibilities' to 'to do's'. Suggests changing 'religious' to 'spirituality'.	
	2	Suggests 'I was unwell' instead of 'my crisis'. Suggests 'things I want to get back to or start' instead of 'things I want to get back to'. Suggests 'fun' instead of 'leisure'. Suggests 'health' as an 'Other' example presumably to broaden options outside of just the religious examples.	

	3	-	
	4	-	
	5	Like the page - great self plan. Suggests removing 'Friends' from 'Friends and social activities' as there should be no assumptions. For the same reason, suggests adding 'friends' to 'Family responsibilities'. Look forward, what's next.	
	6	Notes, responsibilities is a heavy word but thinks it should remain. Suggests 'things I value' instead of responsibilities. Notes, the words 'I value' disappears because of the colour. Notes, doesn't mind the page, not too bad.	
13	1	Notes the participants may not 'just be getting back'.	
	2	-	
	3	Suggests omitting 'for getting back'. Suggests 'Have a plan going forward'.	
	4	-	
	5	Suggests page be completed with a support worker. Likes 'The support I need' as it signals to the consumers it 'isn't assumed to be all on you'.	
	6	-	
14	1	-	
	2	-	
	3	Suggests omitting 'for getting back'. Suggests 'Have a plan going forward'.	
	4	-	

	5	-	
	6	Suggests the colour of the green font should be changed so it's more visible. Suggests bigger boxes so there is more space to write.	
15	1	The page should be earlier in the booklet.	
	2	-	
	3	Agrees - ok	
	4	-	
	5	Suggest the page should be later in the booklet. Notes, the answer to the exercise could be dependent on where the consumer is at in their journey. They think the exercise is a good question - new tools in place, if you're not ready to answer here and now.	
	6	Suggests including a prompt to get the consumer started and include a list of questions. Suggest a peer support worker should help the consumer complete the page.	
16	1	-	
	2	-	
	3	-	
	4	-	
	5	May want to cover more info?	

	6	Like the page - “definitely”	
17	1	-	- 33% participants like the design - colours
	2	-	
	3	-	
	4	Likes the title of the page - short and positive. Likes the design - finds the colours soothing and peaceful.	
	5	-	
	6	Loves the design - colour. Suggest text should be bigger and further up. Perhaps darker blue that was at the beginning of the booklet, to use matching tones.	
18	1	Likes the page.	- 66% participants like the page
	2	Views or suggests that the page should be positive and/or hopeful and empowering. Suggests ‘like my own space’ instead of ‘loner’. Questions use of ‘pessimistic’. Suggests ‘quick to make decisions’ instead of ‘impulsive’. Suggests ‘willingness to express opinions/views.	
	3	Likes the page.	
	4	Dislikes reflecting on personality and categorizing themselves in other people’s views, doesn’t understand the purpose. Worries what the support worker would think of them and doesn’t want to be judged.	
	5	Likes the page - “Nice to recognize who you are when you’re well’ and likes all items. Questions the literacy level of the page?	

	6	Likes the page - “helpful”	
19	1	Likes the page.	- 50% participants like the page
	2	Questions the use of tick boxes and whether there should be so many.	
	3	Suggest exercise needs more explanation and to include lots of examples. Suggested inclusion of spiritual and atheist.	
	4	Likes the page. Likes the life role examples as it reengages the consumer with a feeling of purpose in life. Doesn’t like the occupational examples.	
	5	Likes the page. Notes they would complete the page with the consumer. We are more than that.	
	6	Likes the page but suggests changing a few of the words - son/daughter, brother/sister, man/other - gender capacity, omit religions and replace with a word that is all encompassing.	
20	1	Notes ‘every day’ may not be accurate.	
	2	-	
	3	-	
	4	Questions how this page is different from the previous page. Notes, they wouldn’t have this in their arsenal when unwell. Believes they would need someone with them to talk them through the exercise/do the exercise. Listen to the recording for clarity.	
	5	Likes this page - encourages the consumer to reflect back on the crisis and likes that it is expanding on the journey. May need others to support you, box below, don’t want to feel you’re doing this alone.	
	6	Suggests including tick box prompts of generalised things that people do, split box, line or two	

21	1	-	
	2	-	
	3	-	
	4	Likes that the exercise is an expansion of the previous exercise/page and builds up capacity.	
	5	Likes the page - no pressure	
	6	Suggests including tick box prompts of generalised things that people do, split box, line or two	
22	1	Dislikes ‘avoid’.	- 33% participants like exercise/page
	2	-	
	3	-	
	4	Likes that the exercise/page is an expansion of the previous exercise/page. Suggests completing exercise with a support worker to assist with teasing this out more (e.g. what would help you change this habit? consumer may not have knowledge or insight of what would help).	
	5	Likes the exercise/page.	
	6	Suggests including tick box prompts of generalised things that people do, split box, line or two	
23	1	Suggests ‘calendar’ instead of ‘timetable’. Suggests expanding on instructions e.g. this is just an example, feel free to use your own diary.	- 50% participants like the use of the timetable
	2	-	

	3	-	
	4	Likes the use of the timetable. Notes exercises are easier to build on with the visual of a timetable. Suggest consideration of including the timetable earlier in the workbook or mention earlier that the consumer will have the opportunity to put their work on a timetable. Suggest including a checklist - did I achieve this or not?	
	5	Likes the use of the timetable. Notes, it's similar to NDIS plan. However, it may be sad for the consumer to see less activities.	
	6	Likes the use of the timetable. Notes, could elicit feelings of either 'that's a lot' as a positive or "I have a lot to do in a week". Design - suggests decreasing height and increasing width of the boxes.	
24	1	-	
	2	-	
	3	-	
	4	-	
	5	-	
	6	Likes the page - "good"	
25	1	-	- 33% participants suggest rewording 'Managing ups and downs"
	2	Suggests 'Managing life's ups and downs' and/or 'Moving on Part 2'.	

	3	-	- 33% participants like the colour of the page.
	4	Suggest ‘Managing big feelings’ instead of ‘Managing ups and downs’	
	5	Likes the design - colour.	
	6	Likes the design - “nice, good colours”	
26	1	Suggests ‘What I will do’ instead of ‘the action I will take’.	- 33% participants suggest providing an explanation of what is a trigger and provide examples.<br>- 33% participants suggest rewording ‘the action I will take”
	2	-	
	3	Suggest providing an explanation of what is a trigger and include examples	
	4	Suggests ‘Plan to minimise’ instead of ‘The action plan I will take’.	
	5	Suggest providing an explanation of what is a trigger and include examples	
	6	Design - suggests ‘triggers’ is in red and bold to make it stand out similar to the presentation of ‘action’, reformat to have the trigger and action in the same box.	
27	1	Suggests early warning signs might be better before triggers.	
	2	-	
	3	-	
	4	-	
	5	Suggest to omit this pages as they believe two pages of triggers is not needed and reinforces negativity.	

	6	Design - suggests 'triggers' is in red and bold to make it stand out similar to the presentation of 'action', reformat to have the trigger and action in the same box.	
28	1	-	
	2	-	
	3	Likes 'early warning signs' exercise, believes itself explanatory. Alternatively, they suggest my 'red flags'.	
	4	Suggests early warning signs exercise would be helpful to complete with a support worker as the answer requires a lot of insight. Notes that regarding the action exercise, the consumer may not be able to take an action e.g. domestic violence.	
	5	Likes the page/exercise - likes the use of plain simple language.	
	6	Design - likes that 'early warning signs' and 'action' are bold, reformat to have the trigger and action in the same box.	
29	1	Dislikes 'breaking down' and suggests 'near' instead of 'looming'. Suggest a flow diagram illustrating the recovery phases (see page for example).	- 66% participants suggest the prompt 'breaking down' be reworded.
	2	-	
	3	Suggests 'deteriorating or worsening' instead of 'breaking down', as the latter seems emotionally loaded	
	4	Suggests exercise would be helpful to complete with a support worker as a reflective discussion would be helpful. Similar to previous/early page "feeling worse" - I don't see where the comment was made earlier.	
	5	Likes the page/exercise.	

	6	Suggests ‘changing or escalating’ instead of ‘breaking down’; ‘maybe’ instead of ‘a’. Likes ‘action’. Sense of hope	
30	1	-	
	2	-	
	3	Suggests ‘My Recovery Plan’ instead of ‘My Space’	
	4	-	
	5	Likes the page - “fine”	
	6	-	
31	1	Suggests ‘hopes’ instead of ‘goals’.	
	2	-	
	3	-	
	4	Notes, ‘goals’ can feel like a commitment, presumably a negative. Likes the design - purple is very soothing.	
	5	Likes the page - “excellent”	
	6	Likes page - “always good”	
32	1	Suggests ‘hopes’ instead of ‘goals’.	- 33% participants suggest the consumer would need a support worker to help them complete the exercise.<br>- 50% participants suggest examples should be provided.
	2	-	
	3	-	

	4	Suggests a consumer would need facilitation through the exercises presumably to complete with support worker or providing prompting questions to reflect on/answer and examples.	
	5	Suggests a consumer would need facilitation through the exercises presumably to complete with support worker or providing prompting questions to reflect on/answer and examples.	
	6	Suggests prompts to help the consumer to complete the page. “Oh, no, don’t have goals and dreams” - may elicit that thought or don’t have this section? Design - Don’t have boxes for each goal but provide lines. Suggests using an image e.g. rainbow but notes it could be distracting.	
33	1	Suggests ‘I want to work on’ instead of ‘The goals I want to work on is’’. Suggests it may be too soon for the consumer to formulate a timeframe in regards to ‘How long it might take to achieve this goal’. Suggests omitting goal from ‘My overall plan for working towards this goal’.	- 50% participants suggest the prompt ‘How long it might take to achieve this goal’ be reworded.
	2	-	
	3	Suggests ‘the SMART goal’ and write out acronym - Specific, Measurable, Achievable, Realistic, Timely.	
	4	Dislikes the question ‘How long it might take to achieve this goal’. Suggests instead to ask ‘When is this goal for?’	
	5	Likes the page - simple language`	
	6	Notes ‘How long it might take to achieve this goal’ may be too specific, suggests an approximate time frame to achieve this goal. Likes ‘working towards’. Alternatively, suggests ‘What things I can do (towards) to achieve this goal. Suggests not to add any more boxes as it would be too much writing.	
34	1	Suggests adding a column titled ‘How I might feel when done’.	

	2	Suggests ‘The first steps I could consider’. Suggests removing tick box on completion as it could be empowering but also disempowering.	
	3	-	
	4	Likes that the page breaks down the big goal from the previous page. Loves the tick box when done.	
	5	-	
	6	Suggests including prompts. e.g. Design - reformat boxes to be the same size. Put at the end of each module text from the last chapter.	
35	1	-	
	2	-	
	3	Agrees - good	
	4		
	5	Really likes the page - not assuming you will know how to get the information and from who.	
	6	Design - reformat boxes to be side by side. Suggests providing space for contact info.	
37	1	-	
	2	-	
	3	-	

	4		
	5	-	
	6	Design - realign font to top of leaf, colour is okay though suggests blue would be better or dark grey.	
38	1	Dislikes the recovery quote but doesn't explain what they don't like, suggests use of other examples.	- 60% participants didn't like the quote<br>- 50% participants think the quote should include the author.
	2	Likes the recovery quote but thinks including the author would make it more personable.	
	3	Dislikes quote and that the author is not included. Suggests using a quote from someone "real".	
	4	Really likes the quote - provides context, different things at different times.	
	5	Likes some parts of the quote but not all. Specifically, they think 'I don't let my illness run me' could be triggering. Suggests including space for the consumer to write their own quote.	
	6	Needs an idea. Believes the quote doesn't reflect the concept of recovery. Likes 'driving seat of my life', 'illness' put them off, 'relationship with god' closed them off from doing this. Immediately reacted with wondering who the author is. Really good concepts of recovery.	
39	1	Suggests use infographics, images and icons - less text.	- Content should be more concise. Use less text, more images (infographics, images, icons) to communicate information quickly and clearly.<br>- Alternatively, incorporate the content into the main document.
	2	Suggests "Recovery is about growth - accepting and moving beyond what has happened to you' to 'Recovery is about growth - accepting and moving beyond what I have experienced'. Suggests 'Recovery is about taking back control over your destiny, your issue, your life and the help you need to live it the way you want' instead of 'Recovery is about taking back control over your destiny, your problems, your life and the help you need to live it the	

		way you want'.	
	3	Likes all of the information however suggests making the information more concise, as they question if a consumer would read it entirely. Suggests use of flow chart.	
	4	-	
	5	At no point, a link to this. The length of this section could be frustrating - 4 printed pages. The section should include appropriate content or key elements at each chapter.	
	6	Design - suggests adding a column for notes, dislikes layout (3 columns too much). Suggests incorporating content into main document. Notes, flicking back and forth between pages could cause one to lose concentration/disconnect. A lot of work to x with each month.	
40	1	-	
	2	-	
	3	-	
	4	-	
	5	Suggests to re-order the content in the booklet. 'Using this guide' section should be at the beginning.	
	6		

User Testing Report Phase 2

My Personal Recovery Plan – Digital Prototype

Overall Recommendations

- **Design**
  - Colour
    - Use the current colours but ensure it less washed out
    - Provide a colour customisation toggle so colour can be changed
    - Landing page colour to be less clinical
  - Animations wanted/acceptable
  - Make each drawing/animation consistent with the avatars
  - Size of text for titles made bigger

- **Language**
  - Change the title (to MyPREP)
  - Chunk and reduce words where possible

- **Content**
  - Avatars are highly acceptable, need to ensure avatar options are inclusive to all
  - Need help now button viewed as very important, the emergency services must be easily visible and logos added to aid identification.
  - Accessibility features (eg. voice recording option, read-out loud) highly acceptable

- **Bug fix**
  - Plant animation for small screens
  - Photo attachment capability
  - Logic fixes
  - Supporters

- **Future features/functions**
  - Calendar
  - Restrict the supporter from viewing specific things. (Whole entries, sections, etc.)

- Enabling dashboard rearrangement and renaming capability

Digital prototype Summary Notes by Participant

Page	Participant 1	Participant 2	Participant 3	Participant 4
**Landing Page**	• No major comments	• Does not like the main colour, blue seems too clinical.<br>• Logo needs changing, to be more nature-orientated.	• Blurb in the landing page is quite long and may need to be cut down.<br>• The blue theming to the landing page makes it seem clinical, suggests a brighter colour.<br>• Plant animation is not shown correctly in smaller screens.	• The landing page is very simple, almost too simple. Could use extra colour to draw more attention for first-time users.<br>• Plant animation is not shown correctly in smaller screens.
**Need Help Now!**	Not tested	• Not tested. P2 raised the fact that the tool needs a safety button. Suggested putting help and support as a permanent button on the fixed navbar.	• Condense the view as it does show quite a lot of information.	• Add logos to their respective services.<br>• Make Counselling and Support more prominent.<br>• Possible reword for the button.<br>• Need more direction in the wording to show how users can call more services. (Press here…, use this number to call…)
**Creating a Recovery Plan / Avatars**	• Took 2-3 attempts to create a recovery plan. Attempted to press "View" as there were no	• My Recovery Plan (Possibly changing the name)	• Suggested the voice-over of avatar to be neutral and not overly negative/positive.	• Making the title bigger.<br>• Bullet points aren't showing where they're supposed to be showing.

	obvious hints on where to press to create a recovery plan.<br>• Avoid chunks of text as it does tend to make users less inclined to pay attention and read the text. This leads into the positive reception on using avatars to read blocks of text for the user.<br>• Renaming Avatar/Transcript in the entry screens to something more clear. (Read For Me/Let Me Read)	• Additionally making My Recovery Journal a brighter orange.	• Asked if the Listen or Read should be default (Currently read is default), cases may vary as different personas prefer different mediums.<br>• People may get anxious when recording their voice.	• The plant avatar should be the default view.<br>• The record and text icon buttons to be animated to fit with the design style of the avatars.<br>• When attaching photos in the text entry they appear in full size, overlapping the confines of the text box.<br>• Allowing colour change on the WYSIWYG.
**Dashboard (Post creating a Recovery Plan)**	• Making it clearer that pressing the "Recovery Plan" button will create a new entry. Currently it only shows previous entries and view (Just displaying view reduced the clarity of what it initially does)	• Colours seem fogged out, and when a user in crisis, the colours would draw them away rather than attract them.<br>• Possibly include a self-care tip box in the dashboard.<br>• Commented about changing the order of the chapter icons in the dashboard.	• Participant 1 naturally gravitated towards adding Emergency Contacts/Supporters after completing their recovery plan rather than completing an entry in a section.<br>• They thought emergency contacts will be seen on the page rather than pressing a button to see the list.<br>• On the recent entries box, the eye icon did not	• Suggested adding a calendar where users can plan their appointments with either their supporters or personal appointments.

			immediately signify that the user is able to view it.<br>• Possibly re-orientate the dashboard box to provide a clearer workflow. (Add extra hopscotch steps)	
**Moving After a Crisis (Attempting their first entry)**	• When completing an entry, making the back/next buttons a bit more prominent.<br>• Would rather type their response opposed to recording their response, although sees value.<br>• Attached emoji's which was viewed positively.	• Reduce the amount of text on the page.<br>• Didn't like going back and forth to read the information.<br>• Liked the idea of a voice recording but that was not the first option to come in mind when attempting to enter a response.<br>• Really liked the ability to add images and emoji's in a text entry, to help the user further express their responses.	• Graphics are blurry on smaller screens.<br>• Suggested integrating voice entry within the textbox so the user does not have to focus on picking either voice recording or text entry.<br>• They attempted to press on the steppers to go back but it does not work.<br>• Must "ADD ROW" to confirm the day of the week. Logic fix.	
**Previous entries / "Proceeding to next entry" Screen**		• Putting the chapter icons on the entry pages for further aesthetic.	• When reviewing the previous entry, the participant took quite a while to find the button to "CREATE A NEW ENTRY", could be placed at the top rather than on the bottom.	• Clearer text to signify what section the user has completed. Changing the word "modules" to "sections.
**Supporters**	• P1 liked the way that it was designed to add	• P2 loved the idea of a supporter being able to	• Allowing the user to restrict the supporter from	• Potentially move the layout of the dashboard so Emergency

	supporters. P1 though it was simple and straight-forward. Although when viewing supporters P1 needed help finding where she could manage (delete, view) her supporters.	assist a user in their entries. P2 needed help to find the window to view/delete supporters.	viewing specific things. (Whole entries, sections, etc.)	Contacts/Supports are the main point of interest for the user.
