# Supplementary material for: Co-Production of a Flexibly Delivered Relapse Prevention Tool to Support the Self-Management of Long-Term Mental Health Conditions: Co-Design and User Testing Study
Source: JMIR Form Res. 2024 Feb 23;8:e49110. doi: 10.2196/49110 (PMC10926903; doi:10.2196/49110)
Supplement: Multimedia Appendix 2 [file formative_v8i1e49110_app2.pdf]

For crisis support call **000** in an emergency or Lifeline on **13 11 14**.

MyPREP

NEED HELP NOW?

My Recovery Plan Last Updated: 27/07/2022

CREATE NEW PLAN + PREVIOUS ENTRIES

Moving on after a Crisis

Keeping Well

Managing Up and Downs

Goals and Dreams

My Emergency Contacts

ADD A CONTACT +

Test test epys@sydney.edu.au 0444444444

My Supporter Circle

ADD A SUPPORTER +

Recent Entries

View All Entries

Managing Up and Downs - How will I know when things are breaking down? 19/04/2023 View Entry

Managing Up and Downs - What are my early warning signs? 19/04/2023 View Entry

Managing Up and Downs - What are my triggers? 19/04/2023 View Entry

I need help now!

In an emergency where you or someone you know is in immediate danger or at risk of harm, please contact emergency services on 000 or go to a hospital emergency department.

Counselling and Support

If you or someone you know is experiencing mental distress there are crisis support services who can help.

Lifeline

24-hour Australian crisis counselling service

P: 13 11 14

Lifeline 13 11 14

Suicide Call Back Service

24-hour Australian counselling service

P: 1300 659 467

Suicide Call Back Service

BeyondBlue

24-hour phone support and online chat service and links to resources and apps

P: 1300 22 4636

Beyond Blue

Other Support Services

There are also many other organisations that can support you based on your unique situation. They are there to help you so please do reach out for support.

Mental Health Line

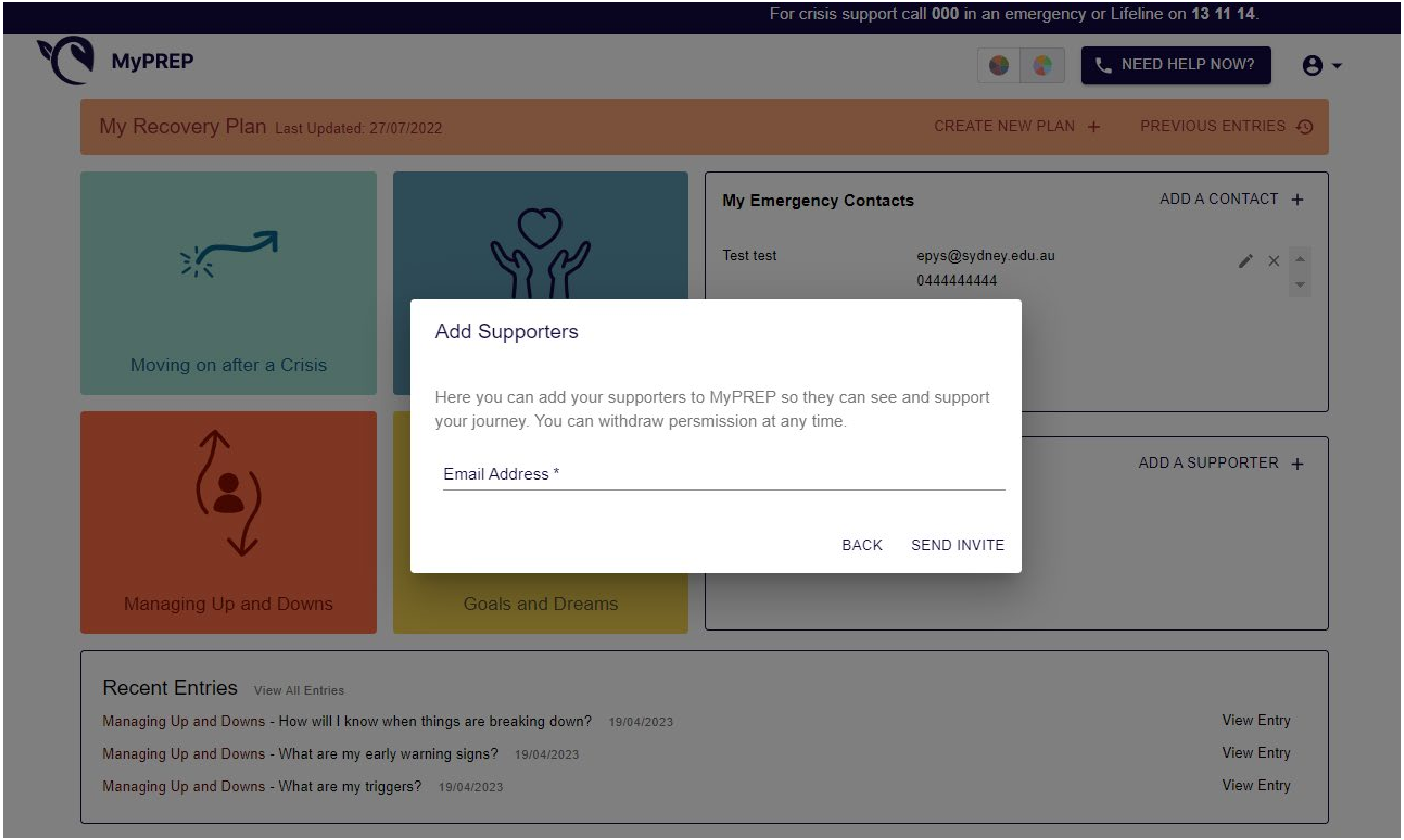
For crisis support call 000 in an emergency or Lifeline on 13 11 14.
MyPREP
NEED HELP NOW?
My Recovery Plan Last Updated: 27/07/2022
CREATE NEW PLAN +
PREVIOUS ENTRIES
My Emergency Contacts
ADD A CONTACT +
Test test
epys@sydney.edu.au
0444444444
Moving on after a Crisis
Add Supporters
Here you can add your supporters to MyPREP so they can see and support your journey. You can withdraw persmission at any time.
Email Address *
BACK
SEND INVITE
ADD A SUPPORTER +
Managing Up and Downs
Goals and Dreams
Recent Entries
View All Entries
Managing Up and Downs - How will I know when things are breaking down? 19/04/2023
View Entry
Managing Up and Downs - What are my early warning signs? 19/04/2023
View Entry
Managing Up and Downs - What are my triggers? 19/04/2023
View Entry

For crisis support call **000** in an emergency or Lifeline on **13 11 14**.

MyPREP

NEED HELP NOW?

Moving on again after a crisis

Please select, to create a new entry

Getting back into day to day routines
Last Updated: 01/08/2022

What will you do if things get worse
Last Updated: 01/08/2022

Things that need sorting out after your crisis
Haven't updated yet

Getting back to the things you did before your crisis
Last Updated: 01/08/2022

What have you learned from this crisis?
Haven't updated yet

LISTEN READ

Getting back into day to day routines

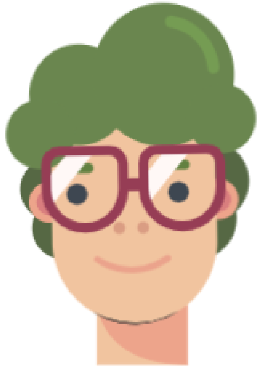

00:02 01:21

Getting into a regular routine as soon as possible can help to stabilise your life again, and many routines may be quite simple, like:

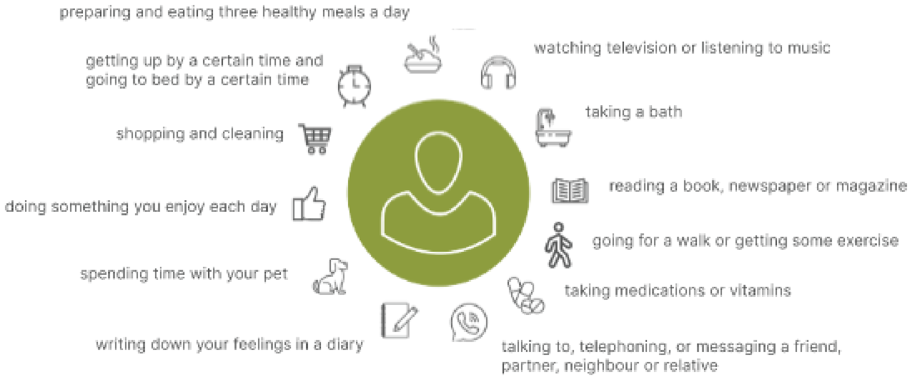

You might also be able to get ideas by talking things through with a mental health worker, relative or friend. Some things you will do on a everyday and other things you will do less often (only on some days each week). You might also want to think about the things you should avoid (like alcohol and drugs or getting over-tired).

We have left space for you to write these things down on the next page. Some people find it helpful to draw up a weekly timetable to get themselves back into a routine and then gradually add more things as they feel up to it.

Introduction - Getting back into day to day routines

Things to do every day to help my life after a crisis

Things to do every day to help my life after a crisis

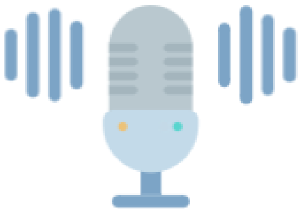

Record your thoughts

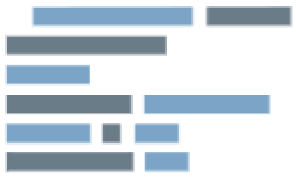

Type your thoughts

BACK NEXT

Things to do on some day(s) each week after a crisis and which days I will do them on

Things to avoid whilst I am recovering from a crisis

Review
